# An Evaluation of the Anti-Carcinogenic Response of Major Isothiocyanates in Non-Metastatic and Metastatic Melanoma Cells

**DOI:** 10.3390/antiox10020284

**Published:** 2021-02-13

**Authors:** Melina Mitsiogianni, Sotiris Kyriakou, Ioannis Anestopoulos, Dimitrios T. Trafalis, Maria V. Deligiorgi, Rodrigo Franco, Aglaia Pappa, Mihalis I. Panayiotidis

**Affiliations:** 1 Department of Applied Sciences, Northumbria University, Newcastle Upon Tyne NE1 8ST, UK; melina.mitsiogianni@northumbria.ac.uk; 2 Department of Electron Microscopy & Molecular Pathology, The Cyprus Institute of Neurology & Genetics, Nicosia 2371, Cyprus; sotirisk@cing.ac.cy (S.K.); ioannisa@cing.ac.cy (I.A.); 3 The Cyprus School of Molecular Medicine, P.O. Box 23462, Nicosia 1683, Cyprus; 4 Laboratory of Pharmacology, Medical School, National & Kapodistrian University of Athens, 11527 Athens, Greece; dtrafal@med.uoa.gr (D.T.T.); mdeligiorgi@yahoo.com (M.V.D.); 5 Redox Biology Centre, University of Nebraska-Lincoln, Lincoln, NE 68583, USA; rodrigo.franco@unl.edu; 6 Department of Veterinary Medicine & Biomedical Sciences, University of Nebraska-Lincoln, Lincoln, NE 68583, USA; 7 Department of Molecular Biology & Genetics, Democritus University of Thrace, 68100 Alexandroupolis, Greece; apappa@mbg.duth.gr

**Keywords:** isothiocyanates, sulforaphane, iberin, allyl isothiocyanate, benzyl isothiocyanate, phenethyl isothiocyanate, anti-cancer agents, melanoma, reactive oxygen species, glutathione, cell cycle arrest, apoptosis, necrosis

## Abstract

Malignant melanoma is one of the most deadly types of solid cancers, a property mainly attributed to its highly aggressive metastatic form. On the other hand, different classes of isothiocyanates, a class of phytochemicals, present in cruciferous vegetables have been characterized by considerable anti-cancer activity in both in vitro and in vivo experimental models. In the current study, we investigated the anti-cancer response of five isothiocyanates in an in vitro model of melanoma consisting of non-metastatic (A375, B16F-10) and metastatic (VMM1, Hs294T) malignant melanoma as well as non-melanoma epidermoid carcinoma (A431) and non-tumorigenic melanocyte-neighboring keratinocyte (HaCaT) cells. Our aim was to compare different endpoints of cytotoxicity (e.g., reactive oxygen species, intracellular glutathione content, cell cycle growth arrest, apoptosis and necrosis) descriptive of an anti-cancer response between non-metastatic and metastatic melanoma as well as non-melanoma epidermoid carcinoma and non-tumorigenic cells. Our results showed that exposure to isothiocyanates induced an increase in intracellular reactive oxygen species and glutathione contents between non-metastatic and metastatic melanoma cells. The distribution of cell cycle phases followed a similar pattern in a manner where non-metastatic and metastatic melanoma cells appeared to be growth arrested at the G2/M phase while elevated levels of metastatic melanoma cells were shown to be at sub G1 phase, an indicator of necrotic cell death. Finally, metastatic melanoma cells were more sensitive apoptosis and/or necrosis as higher levels were observed compared to non-melanoma epidermoid carcinoma and non-tumorigenic cells. In general, non-melanoma epidermoid carcinoma and non-tumorigenic cells were more resistant under any experimental exposure condition. Overall, our study provides further evidence for the potential development of isothiocyanates as promising anti-cancer agents against non-metastatic and metastatic melanoma cells, a property specific for these cells and not shared by non-melanoma epidermoid carcinoma or non-tumorigenic melanocyte cells.

## 1. Introduction

Malignant melanoma (MM) is one of the deadliest types of malignancies, and it is characterized by continuously increasing rates worldwide [1,2,3,4,5]. Although it accounts for approximately 5% of skin cancer cases, it is related to high mortality rates which account for approximately 80% of skin cancer deaths [6]. The aggressiveness of MM is related to low 5-year survival rates (i.e., less than 15%) while patients with advanced stage MM (i.e., stage IV) have a median survival of less than one year [6,7]. MM arises from the progressive accumulation of melanocytic lesions while important risk factors include genetic predisposition as well as prolonged UV exposure, strongly associated with the onset and progression of the disease [8,9,10]. A number of genetic abnormalities have been identified and associated with the onset of MM including mutations in *BRAF*, *NRAS* and *KIT* while additional mutations in *TP53*, *TERT* and *PTEN* are required for the occurrence of the invasiveness of the disease [11,12,13,14]. Currently, treatment options for early stages of MM include surgical resection while systemic therapy (i.e., chemotherapy (e.g., paclitaxel, temozolomide), immuno-therapy by means of cytotoxic T-lymphocyte-associated protein 4 (CTLA-4) and programmed cell death protein 1 (PD-1) inhibitors (e.g., Ipilimumab, Nivolulab, Pembrolizumab) and targeted therapy by means of BRAF and MEK inhibitors (e.g., Dabrafenib, Vemurafenib, Trametinib)) is applied for more advanced and metastatic types of the disease [6,7,11]. However, although the significant clinical progress due to these therapeutic approaches, MM is still incurable as it is associated with high rates of recurrence and poor prognosis. In addition, the observed side effects caused by systemic toxicity, along with drug resistance, can further contribute to the limited therapeutic efficacy against MM [6,11,15]. To this end, the development of new therapeutic approaches is of high importance in order to improve current existing protocols, thereby improving the quality of life in MM patients [6,15,16,17].

**Figure 1:**
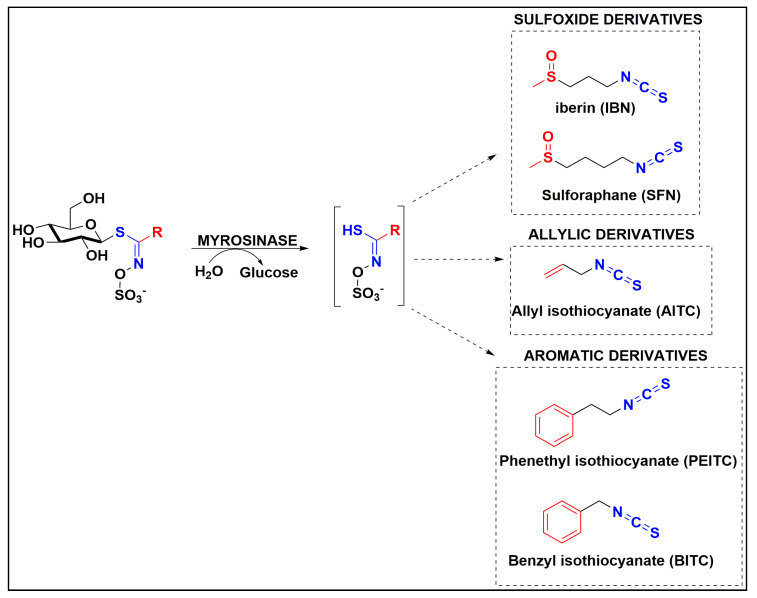
Schematic representation of the enzymatic conversion of glucosinolates into ITCs by the action of myrosinase. Sulfoxide, allylic and aromatic derivatives of glucosinolates are presented as the three major ones along with their respective ITCs namely IBN, SFN, AITC, PEITC and BITC, respectively.

A number of studies have revealed that phytochemicals have been associated with significant anti-cancer activity against a variety of tumors. Many epidemiological studies have indicated an inverse correlation between consumption of fruits and vegetables and risk for cancer development [18,19]. In this context, the beneficiary and health-promoting properties of various phytochemicals against skin malignancies have been extensively described [20,21,22]. Specifically, it has been reported that the consumption of cruciferous vegetables is associated with reduced risk of cancer development, a capacity associated with their high content of sulfur-containing phytochemicals known as isothiocyanates (ITCs) [23,24,25,26]. These are secondary metabolites obtained from hydrolysis of their precursor molecules (glucosinolates), by myrosinase, an enzyme activated after plant tissue disruption [27] (Figure 1). The importance of ITCs as nutraceutical agents is reflected upon their capacity to modulate enzymes involved in (i) detoxification, (ii) apoptotic induction, (iii) cell cycle growth arrest and (iv) interactions with various other cellular pathways of tumor growth and invasion known to be deregulated in various cancers including MM [28,29,30,31,32,33,34,35,36,37,38].

Although the great majority of studies have documented an anti-cancer activity of ITCs against non-metastatic melanoma cell lines [33,34,35], their effect in metastatic melanoma cells has remained largely undetermined. Thus, in the present study, we have aimed to evaluate and compare the anti-cancer potency of five major ITCs (e.g., sulforaphane; SFN, iberin; IBN, allyl isothiocyanate; AITC, benzyl isothiocyanate; BITC and phenethyl isothiocyanate; PEITC) in metastatic (A375, B16F-10) and non-metastatic (VMM1, Hs294T) melanoma cells. Furthermore, we have adopted non-melanoma epidermoid carcinoma (A431) and immortalized non-tumorigenic melanocyte-neighboring keratinocyte (HaCaT) cells in order to characterize the specificity of our observations to MM alone.

## 2. Materials and Methods

### 2.1. Cell Lines and Cultures

A375 and A431 cells were purchased from Sigma-Aldrich (St. Louis, MO, USA). HaCaT cells were kindly provided by Sharon Broby (Dermal Toxicology & Effects Group; Centre for Radiation, Chemical and Environmental Hazards; Public Health England, UK). Finally, VMM1, Hs294T and B16F-10 cells were obtained from LGC Standards (Middlesex, UK). All cells were authenticated by Short Tandem Repeat (STR) profiling, tested for mycoplasma and cultured for 15–20 passages before the usage of new stocks. All cell lines were maintained in a humidified atmosphere at 37 °C and 5% CO_2_ and according to the provider’s recommended culture conditions.

### 2.2. Exposure Protocols

For the estimation of reactive oxygen species (ROS), glutathione (GSH), cell cycle kinetics, apoptosis and necrosis, cells were seeded in 100 mm dishes, followed by overnight incubation. The next day, all cells were exposed to 10 μM of each ITC for 24 and 48 h incubation periods. Untreated (control) cells were incubated with either 0.1% DMSO or 0.1% EtOH. The density of seeded cells was determined as previously published [16,17].

### 2.3. Determination of Biological Endpoints

After exposure to each ITC, a single cell suspension of 10^6^ cells/mL was prepared. For the determination of ROS, DHR 123 (10 µM) was added in the suspension and incubated for 5 min at 37 °C while DAPI (1 µM) was added to each sample and incubated for additional 5 min, in order to determine the % of dead cells. For the determination of GSH, 5 µL of ThiolGreen detection reagent was added into each cell suspension. Samples were incubated at 37 °C (for 30 min), centrifuged (at 1000 rpm for 4 min) and cell pellets were resuspended in 1 mL of Assay Buffer. Finally, DAPI (1 µM) was added into each sample and incubated for 5 min in order to determine the % of dead cells in the suspension. For the determination of cell cycle kinetics, the FxCycle PI/RNase staining solution was used according to the manufacturer’s instructions. Briefly, about 0.5 × 10^6^ cells were fixed in cold 70% ethanol, for 1 h or longer, and kept at 4 °C until further processing. Then, cells were washed twice with PBS to remove ethanol and finally suspended in FxCycle PI/RNase staining solution for 30 min, at room temperature, in dark conditions. For the detection of apoptosis, the CellEvent Caspase 3/7 Green flow cytometry assay kit was used, according to the manufacturer’s instructions. Briefly, 0.5 µL of CellEvent Caspase 3/7 Green detection reagent was added into 0.5 mL of each cell suspension and samples were incubated at 37 °C for 30 min. Finally, DAPI (1 µM) was added to each sample and incubated for 5 min in order to determine the percent of dead cells in the suspension. Caspase-3/7-positive and DAPI-positive cells were identified as apoptotic and/or necrotic, respectively. For all determinations, 10,000 events were used (for each sample) while data acquisition and analysis were performed using a FACS Canto II flow cytometer (BD Biosciences, San Jose, CA, USA).

### 2.4. Statistical Analyses

Data were expressed as mean values ± standard deviation (SD). Comparisons between control and treated groups were analyzed by one-way ANOVA with Tukey’s test for multiple comparisons using the SPSS v.22 software. Levels of *p* < 0.05, *p* < 0.01 and *p* < 0.001 were considered statistically significant.

## 3. Results

We have previously provided evidence that SFN, IBN, AITC, BITC and PEITC exhibited significant cytotoxic activity against an in vitro model of MM [16,17]. Based on these findings, exposure to 10 μΜ, at 24 and 48 h, of each ITC were selected as the optimum conditions for all experiments in this study. To this end, we have adopted these conditions in order to evaluate if the previously documented cytotoxic capacity of ITCs [16,17] is mediated through perturbations in levels of ROS, GSH, apoptosis, and necrosis as well as changes in cell cycle growth arrest kinetics.

**Figure 2:**
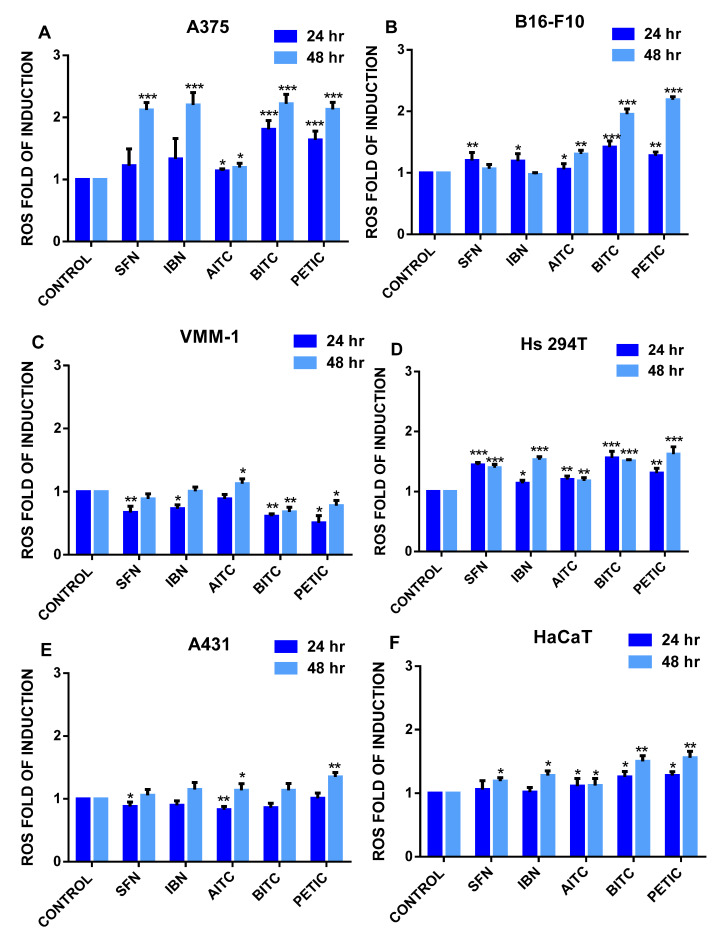
The effect of various ITCs on ROS levels in an in vitro model of MM. Cells were exposed to 10 µM of each ITC, for 24 and 48 h, and monitored by means of flow cytometry. Results were quantitated as ROS fold induction levels for (**A**) A375, (**B**) B16F-10, (**C**) VMM1, (**D**) Hs294T, (**E**) A431 and (**F**) HaCaT cells. Data shown are means ± SD of three replicates from three independent experiments. * *p* < 0.05. ** *p* < 0.01, *** *p* < 0.001, when compared to untreated (control) cells.

For assessing ROS production, we have used a flow cytometry approach (Figure 2A–F and Supplementary Figure S1A–F). Our data revealed that for the majority of ITCs, 48 h of exposure induced higher levels of ROS (when compared to 24 h) in all cell lines. Specifically, the most robust ROS induction was observed in A375 and B16F-10 (Figure 2A,B, respectively) compared to VMM1 and Hs294T (Figure 2C,D, respectively) cell lines. However, A431 and HaCaT cells appeared to be more resistant in ITCs-induced ROS generation (Figure 2E,F, respectively). In fact, their ROS levels were almost similar to those observed in metastatic melanoma cells.

**Figure 3:**
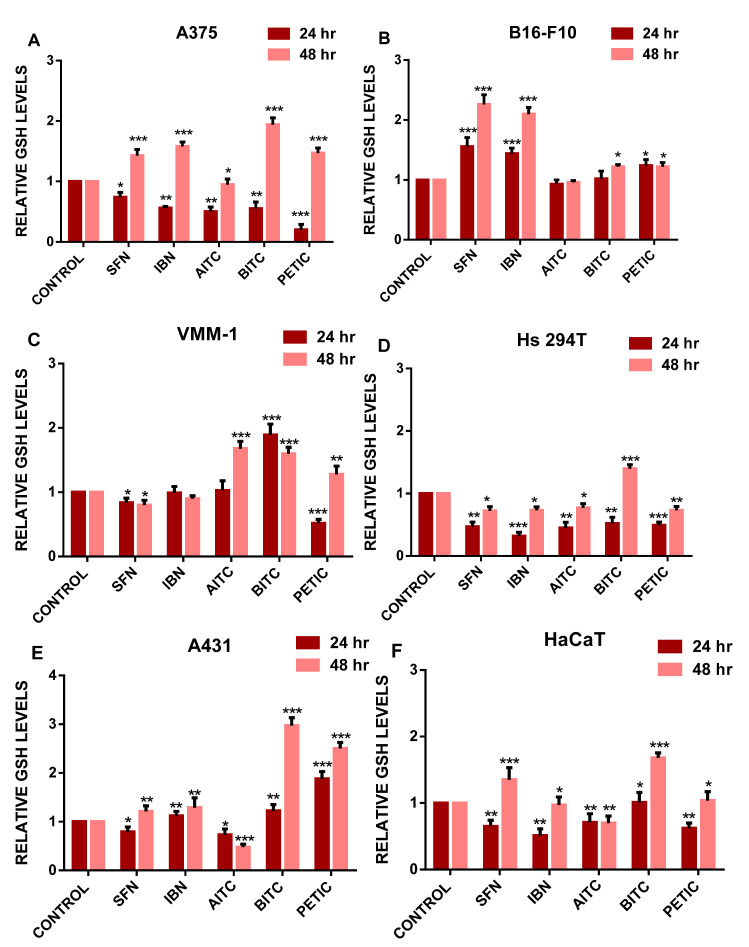
The effect of various ITCs on GSH levels in an in vitro model of MM. Cells were exposed to 10 µM of each ITC, for 24 and 48 h, and monitored by means of flow cytometry. Results were quantitated as relative GSH levels for (**A**) A375, (**B**) B16F-10 (**C**) VMM1, (**D**) Hs294T, (**E**) A431 and (**F**) HaCaT cells. Data shown are means ± SD of three replicates from three independent experiments. * *p* < 0.05, ** *p* < 0.01, *** *p* < 0.001, when compared to untreated (control) cells.

Next, we sought to determine the effects of ITCs on the intracellular levels of GSH by flow cytometry (Figure 3A–F and Supplementary Figure S2A–F). According to our data, the main pattern of GSH levels followed either a significant decrease (Figure 3A,D,F) or remained at control levels (Figure 3B,C,E) over 24 h of exposure for the majority of ITCs, in all cell lines, accompanied by a marked increase at 48 h of ITCs exposure (Figure 3A–F).

**Figure 4:**
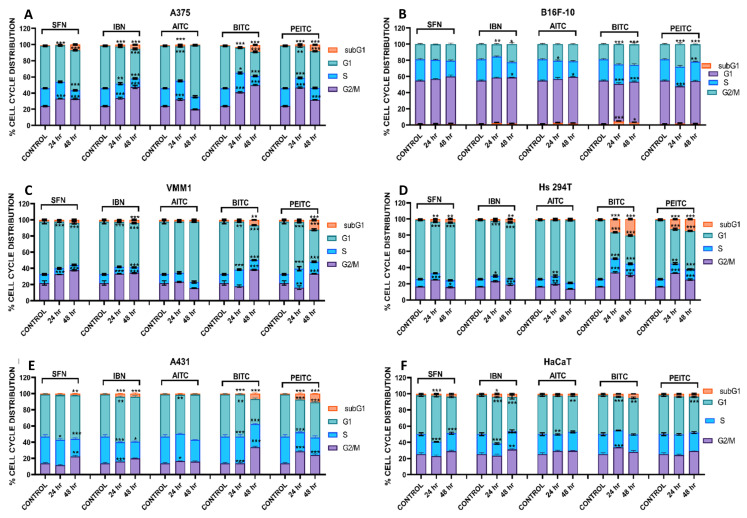
The effect of various ITCs on cell cycle progression in an in vitro model of MM. Cells were exposed to 10 µM of each ITC, for 24 and 48 hr, and monitored by means of flow cytometry. Results were quantified as percent of total DNA content accumulated at each phase of the cell cycle (e.g., sub-G1, G1, S and G2/M) for (**A**) A375, (**B**) B16F-10, (**C**) VMM1, (**D**) Hs294T, (**E**) A431 and (**F**) HaCaT cells. Data shown are means ± SD of three replicates from three independent experiments. * *p* < 0.05, ** *p* < 0.01, *** *p* < 0.001, when compared to untreated (control) cells.

In the next series of experiments, we examined the effect of ITCs exposure in causing perturbations on cell cycle progression by flow cytometry (Figure S3A–F). In A375 and B16F-10 cells, ITCs induced cell cycle growth arrest at G2/M phase, an effect that was intensified after 48 h of exposure, in A375 cells (Figure 4A), while, in B16F-10 cells, this was clearly not the case as there was no evidence of growth arrest (Figure 4B). In VMM1 and Hs294T cells, exposure to ITCs resulted in G2/M growth arrest but to a lesser extent when compared to A375 cells (Figure 4C,D). In A431 cells, ITCs exhibited a G2/M growth arrest evident at 48 h of exposure only while non-tumorigenic cells did not exhibit any evidence of cell cycle growth arrest (Figure 4E,F, respectively). Overall, our findings indicate cell cycle perturbations in non-metastatic and metastatic melanoma as well as non-melanoma cells (with a predominant growth arrest at G2/M phase) while non-tumorigenic cells remained largely unaffected after exposure to ITCs.

**Figure 5:**
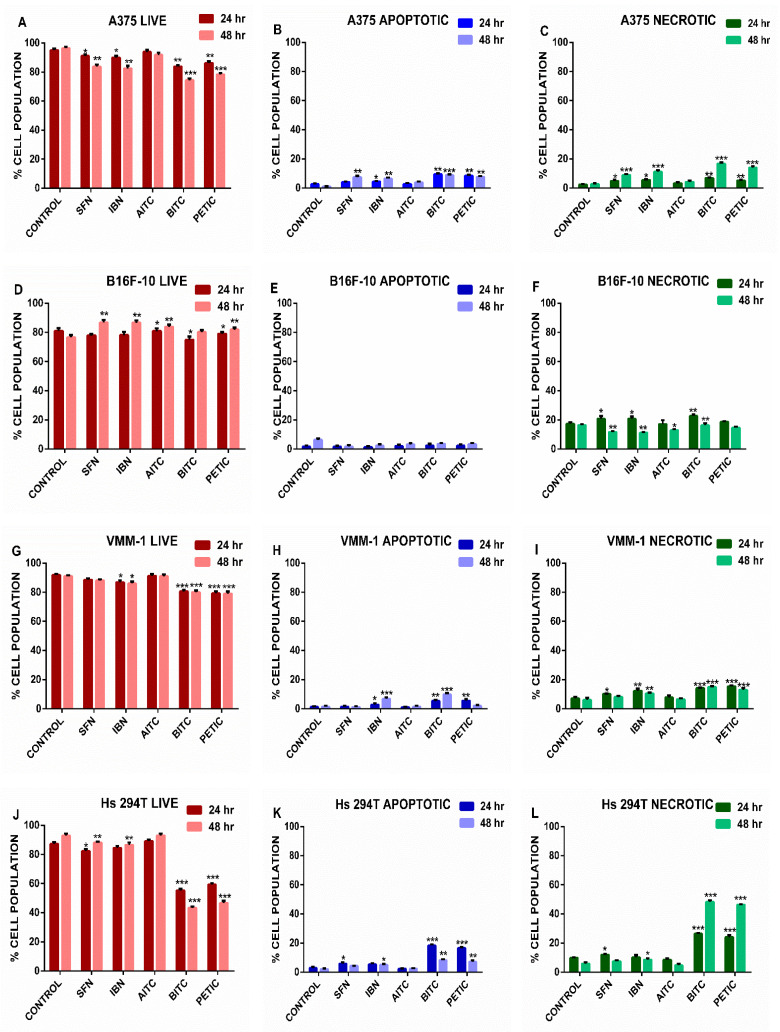

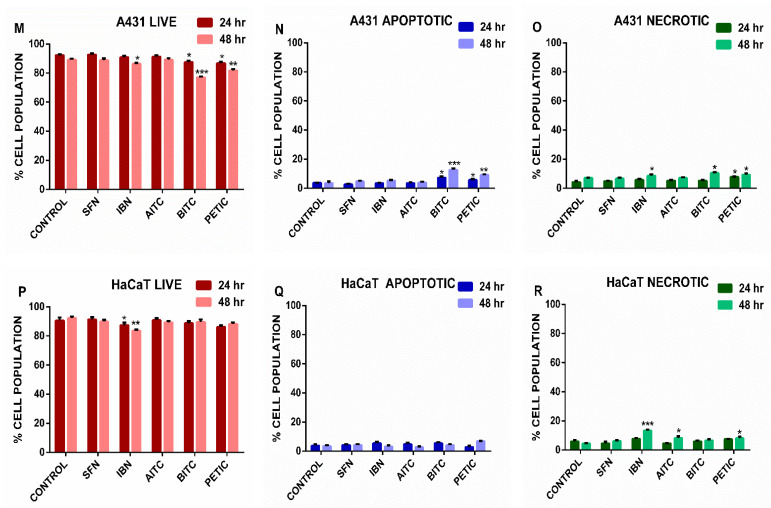
The effect of various ITCs on apoptotic and necrotic cell death in an in vitro model of MM. Cells were exposed to 10 µM of each ITC, for 24 and 48 h, and monitored by means of flow cytometry. Results were quantitated as percent of live, apoptotic and necrotic cell populations for (**A–C;** A375), (**D–F;** B16F-10), (**G–I;** VMM1), (**J–L;** Hs294T), (**M–O;** A431), (**P–R;** HaCaT) cells. Data shown are means ± SD of three replicates from three independent experiments. * *p* < 0.05, ** *p* < 0.01, *** *p* < 0.001, when compared to untreated (control) cells.

Finally, we evaluated the type of cell death under the same experimental conditions by using flow cytometry in order to distinguish between necrosis and apoptosis. Overall, our data indicated that exposure to ITCs exhibited a modest induction of necrosis while minimally affecting apoptosis as the great majority of the various cell types remained viable at the end of each exposure protocol. Specifically, A375 and B16F-10 cells appeared to be more sensitive to necrosis (Figure 5C,F) rather than apoptosis (Figure 5B,E) after exposure to ITCs. These observations were evenly distributed among all tested ITCs. On the other hand, VMM1 and Hs294T cells appeared to follow the same pattern of apoptotic (Figure 5H,K) and/or necrotic (Figure 5I,L) cell death except that the effect of aromatic compounds (e.g., BITC and PEITC) was substantially higher in both types of cell death. Finally, A431 and HaCaT cells showed to be resistant to ITCs-induced cell death as evident by a small percent of apoptotic (Figure 5N,Q) and/or necrotic (Figure 5O,R) cells. Overall, our results indicate that exposure to ITCs induces both a necrotic as well as apoptotic cell death in non-metastatic and metastatic melanoma cells while non-melanoma epidermoid carcinoma and non-tumorigenic cells remain largely unaffected.

## 4. Discussion

MM is one of the most aggressive and lethal types of solid cancers worldwide, with its metastatic form accounting for 80% of all deaths related to skin cancer, despite the use of several promising therapeutic treatment options [6,39]. On the other hand, epidemiological studies revealed that a balanced diet, rich in cruciferous vegetables, is inversely related with the incidence of skin cancer development [23]. To these ends, ITCs represent an important class of bioactive dietary compounds that exhibit a wide range of biological activities including anti-inflammatory, anti-bacterial, anti-aging, and anti-cancer in various types of cancers. In MM, the underlined ITCs-induced anti-cancer mechanisms are mediated through alterations in various otherwise deregulated cellular signaling pathways associated with cell growth, proliferation and apoptosis, thereby negatively regulating the initiation and progression of this type of malignancy [24,25].

Initially, we have evaluated the potential of ITCs to induce oxidative stress as an important parameter of their cytotoxicity. Despite ITCs-induced stimulation of oxidative stress, a differential response in ROS production was evident depending on the cell type itself as well as the class of ITC used in each exposure protocol. Specifically, our results indicated that ROS induction in non-metastatic melanoma cells was significantly higher compared to metastatic melanoma, non-melanoma epidermoid carcinoma and non-malignant cells. The observed differences in contents of intracellular ROS levels could be linked to the differential response of each cell type against ITCs-induced cytotoxicity. Overall, our data are in agreement with other reports indicating that ITCs (particularly BITC and PEITC) can promote ROS-induced cytotoxicity in various in vitro models of lung cancer [40,41,42]. Furthermore, in another study, SFN was shown to increase ROS levels in a p53-null osteosarcoma (MG-63) as well as bronchial epithelial (BEAS-2B) cell lines where ROS accumulation was accompanied by increased expression levels of Nrf2 and heme oxygenase-1 (OH-1) leading to cell death [43,44].

Then, we attempted to evaluate the role of GSH in the differential response of each cell line against exposure to ITCs. Overall, the levels of total intracellular GSH content were significantly increased in non-metastatic cells while the same pattern was observed also in all the other cell lines as well, but to a lesser extent. The marked increase in GSH levels was particularly evident at 48 h of ITCs exposure, an observation that may be interpreted either as a consequence to increased levels of oxidative stress or as an adaptive response for the metabolism of ITCs via the mercapturic acid pathway. For the latter, many reports suggest that the mode of action of ITCs is based on their ability to conjugate with intracellular GSH forming dithio-carbamates [45,46,47,48]. However, these conjugates appeared unstable due to extracellular hydrolysis, resulting in the burst elevation of GSH that is reabsorbed back to the cell [45,49]. To this end, another study has suggested that ITCs are capable of inducing oxidative stress due to their conjugation with intracellular GSH, thereby deactivating a major constituent of the intracellular antioxidant cell defense mechanism [50,51].

Moreover, when assessing cellular distribution in the context of cell cycle growth arrest, metastatic and non-metastatic melanoma cells showed a similar pattern indicative of a G2/M phase growth arrest. In addition, non-melanoma epidermoid carcinoma cells were also shown to be growth arrested at G2/M phase, an observation that was not shared by non-malignant cells as they remained largely unaffected after exposure to ITCs. A number of studies have provided contradicted data regarding the capacity of ITCs to induce growth arrest at G2/M phase in various cancer cell lines [52,53,54,55] or other cell cycle phases like G0/G1 [56] and G1/S [57] as well. In particular, exposure of epithelial colorectal adenocarcinoma (CaCo-2 and SW620) cells to SFN, AITC, BITC and PEITC was shown to induce a growth arrest at G2/M phase while exposure of human oral squamous carcinoma (HSC-3) and breast ductal carcinoma (ZR-75-1) cells to PEITC and SFN respectively caused growth arrest at G0/G1 phase [55,56,57]. To these ends, exposure to IBN also appears to follow the same pattern in neuroblastoma (HT 92 and SK-N/-SH) cells by inducing a growth arrest at G2/M and G0/G1 phases, respectively [58].

Finally, our findings confirmed that ITCs are capable of inducing a modest induction of apoptotic and/or a necrotic cell death, in all cell lines, but to a different extent depending on cell type. Specifically, the levels of apoptosis were diminished when compared to those of necrosis in all cell lines. In addition, levels of both necrosis and apoptosis were significantly higher in metastatic and non-metastatic melanoma cells when compared to non-melanoma epidermoid carcinoma and non-tumorigenic ones. This is of major importance as previous work, by our group, has shown that, although higher concentrations of ITCs (25–100 μM) were capable of exerting significantly higher levels of an anti-cancer response, this was demonstrated by a pattern of non-specificity towards both tumorigenic and non-tumorigenic cells [16,17,26]. In other words, an ITC concentration above 10 μΜ appeared to be considerably more cytotoxic for any cell line regardless if it was tumorigenic or not (i.e., non-specific potency) [16,17,26]. To overcome the barrier of such non-specificity, we have selected an ITC concentration of 10 μΜ, over 48 h of exposure, as this was shown to be the maximum concentration capable of exerting cytotoxicity to melanoma cells while tumorigenic non-melanoma (A431) as well as non-tumorigenic keratinocyte (HaCaT) cells remained relatively resistant [16,17,26]. Finally, according to the literature, the cytotoxic effect of ITCs appears to be mediated through activation of both apoptotic and necrotic cell death, a response that is related to the specific type of cells used under various experimental protocols, thus indicating a bimodal mode of cell death induction [59,60,61,62].

## 5. Conclusions

In the present study, we have provided evidence that major ITC compounds (e.g., SFN, IBN, AITC, BITC and PEITC) exert a differential anti-cancer response against metastatic and non-metastatic melanoma cells, by means of increased intracellular ROS and total reduced GSH levels and perturbations in cell cycle distribution kinetics along with activation of cytotoxicity-induced apoptotic and/or necrotic cell death. Specifically, BITC and PEITC were shown to be the most potent compounds in inducing activation of necrotic cell death in metastatic melanoma cell lines when compared to the non-metastatic ones. At the same time, non-melanoma epidermoid carcinoma and immortalized melanocyte-neighboring keratinocyte cells were shown to be more resistant to treatment with all ITCs. Moreover, our data showed that the order of ITCs’ cytotoxic potency was as follows: BEITC~PEITC > SFN~IBN > AITC. These observations are in agreement with the results from other studies suggesting that the structure of ITCs is strongly associated with their cytotoxic potential as aromatic compounds (BITC and PEITC) appear to be most potent when compared to the aliphatic (AITC) and organosulfur (SBN and IBN) ones [63,64,65]. Furthermore, this effect could be attributed to the relative volatility of ITCs as aromatic ITCs appear to be considerably less volatile compared to allylic ones [66]. In conclusion, our study provides further evidence about the potential of ITCs to act as novel therapeutic agents, thereby supporting their inclusion in pharmaceutical drug development against MM.

## Data Availability

Data are contained within the article and supplementary material.

## Supplementary Materials

The following are available online at https://www.mdpi.com/2076-3921/10/2/284/s1, Figure S1: The effect of ITCs (SFN, IBN, AITC, BITC, PEITC) on ROS levels in an in vitro model of malignant melanoma, Figure S2: The effect of ITCs (SFN, IBN, AITC, BITC, PEITC) on GSH levels in an in vitro model of malignant melanoma, Figure S3: The effect of ITCs (SFN, IBN, AITC, BITC, PEITC) on cell cycle in an in vitro model of malignant melanoma, Figure S4: The effect of ITCs (SFN, IBN, AITC, BITC, PEITC) to induce apoptosis and/or necrosis in an in vitro model of malignant melanoma.
