# Supplementary material for: An Evaluation of the Anti-Carcinogenic Response of Major Isothiocyanates in Non-Metastatic and Metastatic Melanoma Cells
Source: Antioxidants (Basel). 2021 Feb 13;10(2):284. doi: 10.3390/antiox10020284 (PMC7918923; doi:10.3390/antiox10020284)
Supplement: Supplementary file 1 [file antioxidants-10-00284-s001.pdf]

An evaluation of the anti-carcinogenic response of major isothiocyanates in non-metastatic and metastatic melanoma cells

Electronic Supplementary Material

Melina Mitsiogianni[1#], Sotiris Kyriakou[2,3#], Ioannis Anestopoulos[2,3], Dimitrios T. Trafalis[4], Maria V. Deligiorgi[4], Rodrigo Franco[5,6], Aglaia Pappa[7], Mihalis I. Panayiotidis[1,2,3*]

[1] Department of Applied Sciences, Northumbria University, Newcastle Upon Tyne, NE1 8ST, UK; melina.mitsiogianni@northumbria.ac.uk, m.panagiotidis@northumbria.ac.uk
[2] Department of Electron Microscopy & Molecular Pathology, The Cyprus Institute of Neurology & Genetics, Nicosia, 2371, Cyprus; sotirisk@cing.ac.cy, ioannisa@cing.ac.cy, mihalisp@cing.ac.cy
[3] The Cyprus School of Molecular Medicine, PO Box 23462, Nicosia, 1683, Cyprus, sotirisk@cing.ac.cy, ioannisa@cing.ac.cy, mihalisp@cing.ac.cy
[4] Laboratory of Pharmacology, Medical School, National &Kapodistrian University of Athens, Athens, 11527, Greece; dtrafal@med.uoa.gr, mdeligiorgi@yahoo.com
[5] Redox Biology Centre, University of Nebraska-Lincoln, Lincoln, 68583, USA; rodrigo.franco@unl.edu
[6] Department of Veterinary Medicine & Biomedical Sciences, University of Nebraska-Lincoln, Lincoln, 68583, USA; rodrigo.franco@unl.edu
[7] Department of Molecular Biology & Genetics, Democritus University of Thrace, Alexandroupolis, 68100, Greece; apappa@mbg.duth.gr
* Correspondence: mihalisp@cing.ac.cy; Tel.: +357-22392626
[#] Equally contributing authors

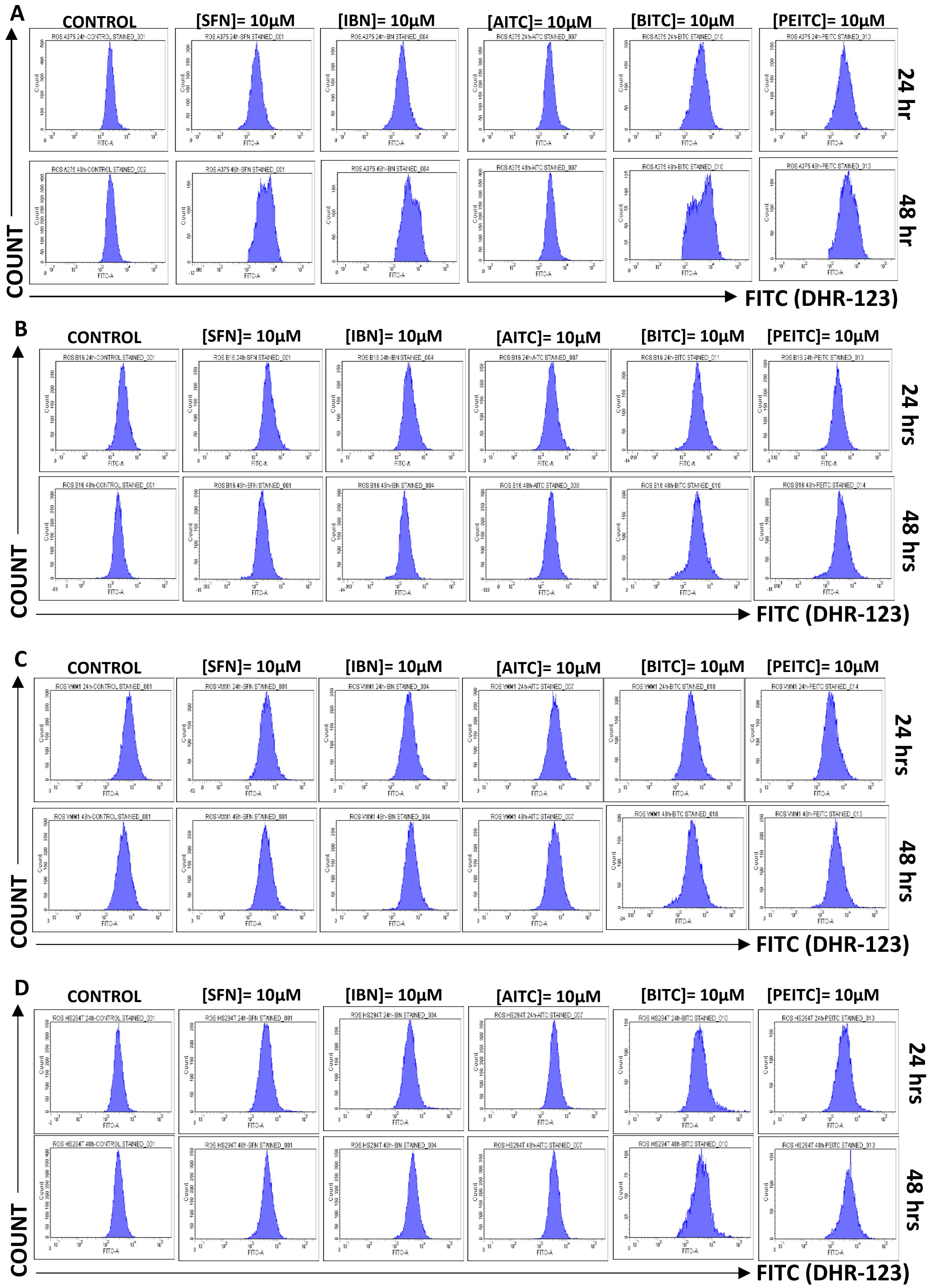

A
CONTROL
[SFN]= 10µM
[IBN]= 10µM
[AITC]= 10µM
[BITC]= 10µM
[PEITC]= 10µM
24 hr
48 hr
COUNT
FITC (DHR-123)
B
CONTROL
[SFN]= 10µM
[IBN]= 10µM
[AITC]= 10µM
[BITC]= 10µM
[PEITC]= 10µM
24 hrs
48 hrs
COUNT
FITC (DHR-123)
C
CONTROL
[SFN]= 10µM
[IBN]= 10µM
[AITC]= 10µM
[BITC]= 10µM
[PEITC]= 10µM
24 hrs
48 hrs
COUNT
FITC (DHR-123)
D
CONTROL
[SFN]= 10µM
[IBN]= 10µM
[AITC]= 10µM
[BITC]= 10µM
[PEITC]= 10µM
24 hrs
48 hrs
COUNT
FITC (DHR-123)

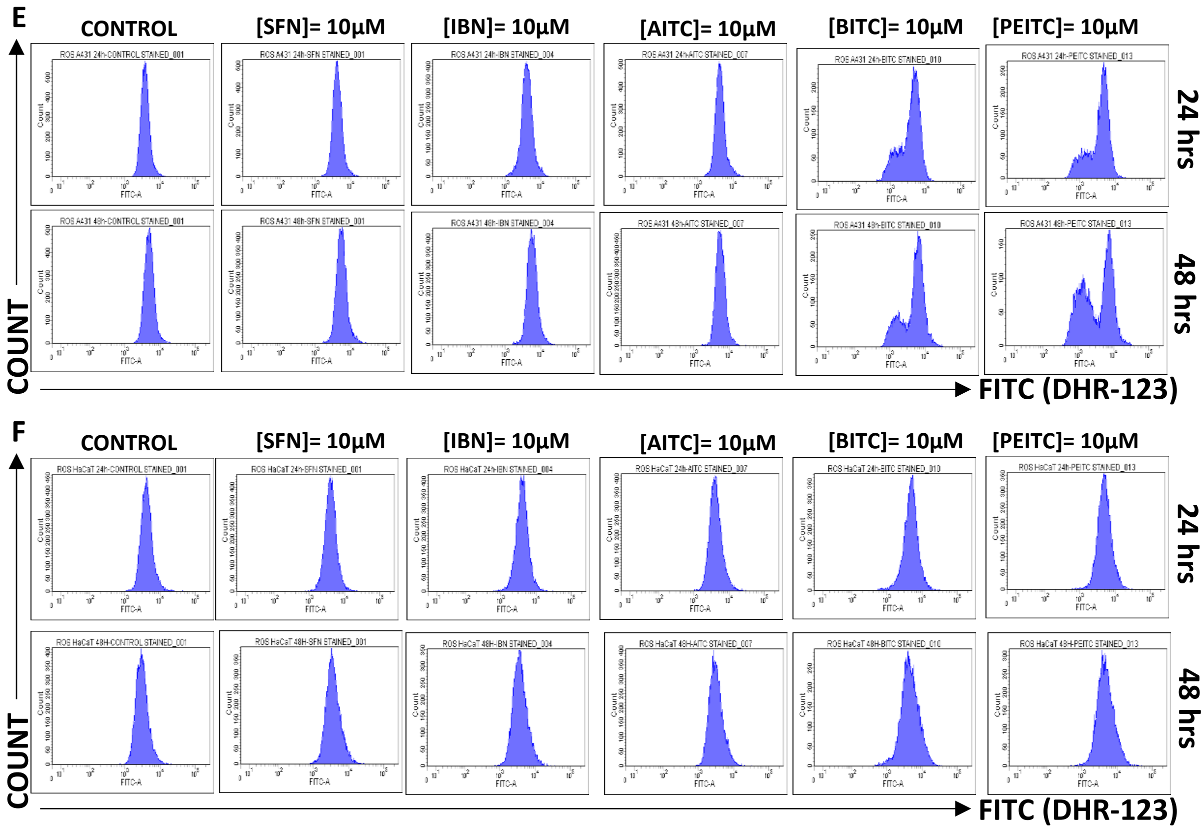

Figure S1: The effect of ITCs (SFN, IBN, AITC, BITC, PEITC) on ROS levels in an *in vitro* model of malignant melanoma consisting of (**A**) human malignant melanoma (A375), (**B**) murine malignant melanoma (B16F-10), (**C**) human brain metastatic melanoma (VMM1), (**D**) human lymph node metastatic melanoma (Hs 294T), (**E**) human non-melanoma epidermoid carcinoma (A431) and (**F**) human immortalized keratinocyte (HaCaT) cell lines. Cells were seeded in a 100 mm dish and next day were exposed to 10 μM of each ITC for 24 and 48 hrs. Treated cells were incubated with the DHR-123 fluorescent probe and ROS levels were analyzed by using a FACS Canto II flow cytometer (BD Biosciences, San Jose, CA, USA). All data were compared to the respective untreated (control) samples. Representative data from three independent experiments.

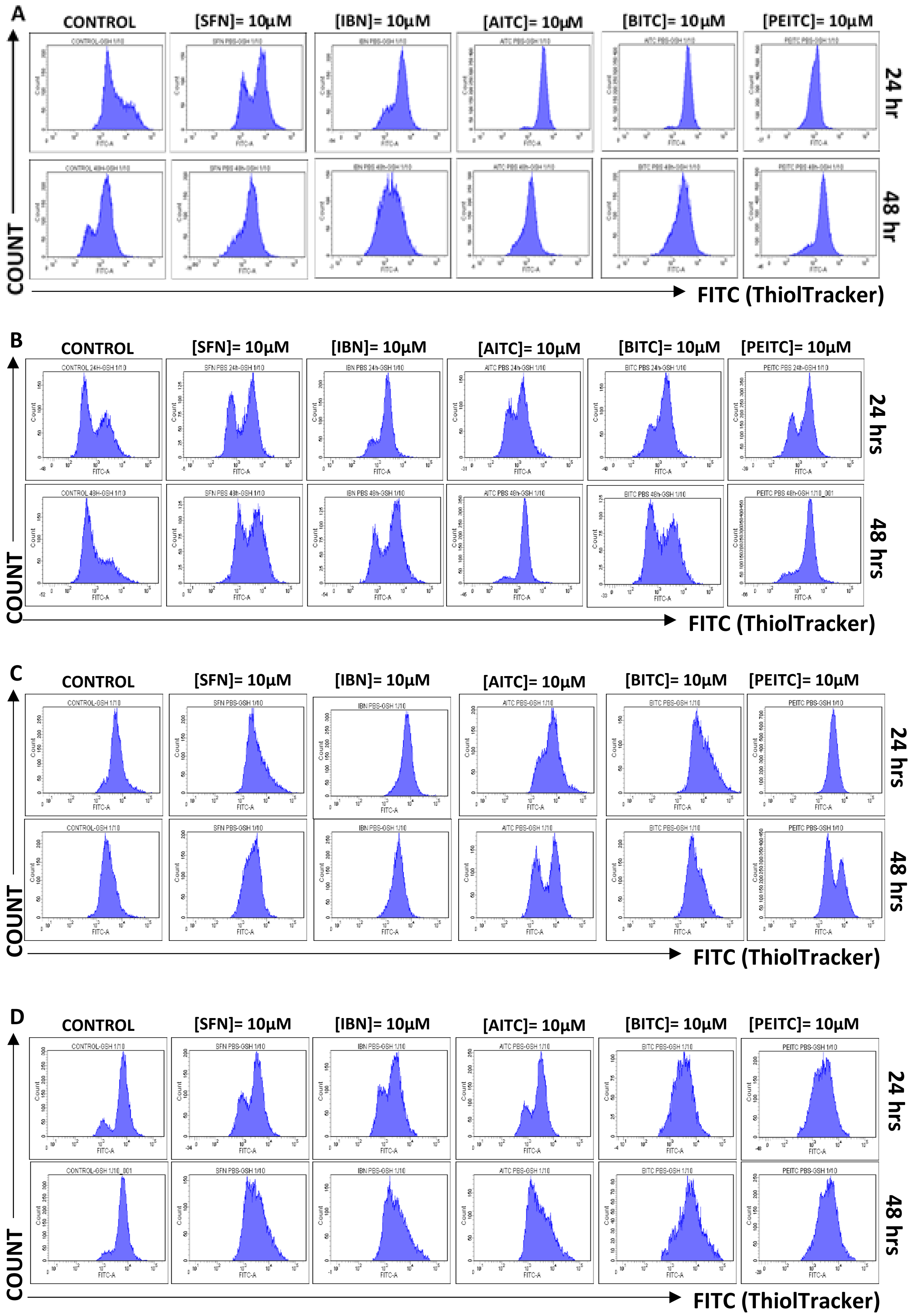

A
CONTROL
[SFN]= 10µM
[IBN]= 10µM
[AITC]= 10µM
[BITC]= 10µM
[PEITC]= 10µM
24 hr
48 hr
COUNT
FITC (ThiolTracker)
B
CONTROL
[SFN]= 10µM
[IBN]= 10µM
[AITC]= 10µM
[BITC]= 10µM
[PEITC]= 10µM
24 hrs
48 hrs
COUNT
FITC (ThiolTracker)
C
CONTROL
[SFN]= 10µM
[IBN]= 10µM
[AITC]= 10µM
[BITC]= 10µM
[PEITC]= 10µM
24 hrs
48 hrs
COUNT
FITC (ThiolTracker)
D
CONTROL
[SFN]= 10µM
[IBN]= 10µM
[AITC]= 10µM
[BITC]= 10µM
[PEITC]= 10µM
24 hrs
48 hrs
COUNT
FITC (ThiolTracker)

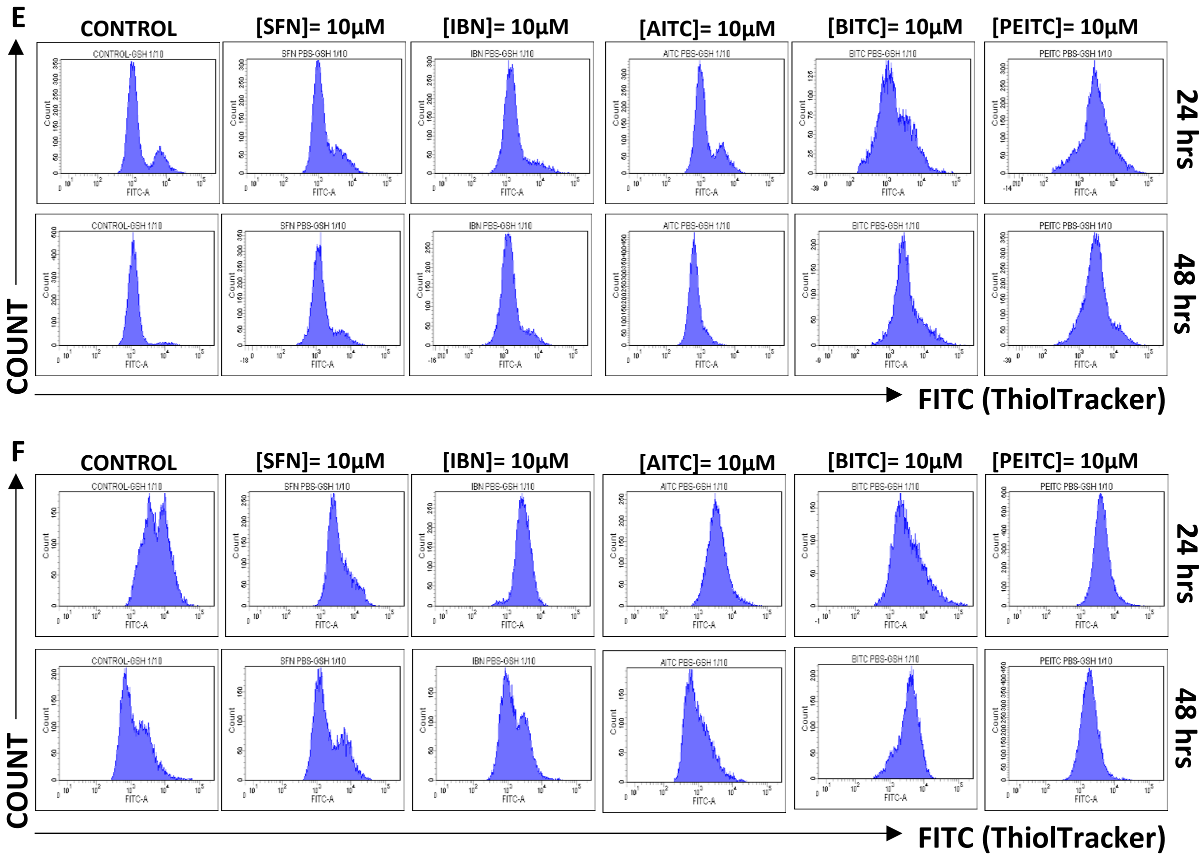

Figure S2: The effect of ITCs (SFN, IBN, AITC, BITC, PEITC) on GSH levels in an *in vitro* model of malignant melanoma consisting of (**A**) human malignant melanoma (A375), (**B**) murine malignant melanoma (B16F-10), (**C**) human brain metastatic melanoma (VMM1), (**D**) human lymph node metastatic melanoma (Hs 294T), (**E**) human non-melanoma epidermoid carcinoma (A431) and (**F**) human immortalized keratinocyte (HaCaT) cell lines. Cells were seeded in a 100 mm dish and next day were exposed to 10 μM of each ITC for 24 and 48 hrs. Treated cells were incubated with the ThiolTracker fluorescent probe and the levels of GSH were analyzed by using a FACS Canto II flow cytometer (BD Biosciences, San Jose, CA, USA). All data were compared to the respective untreated (control) samples. Representative data from three independent experiments.

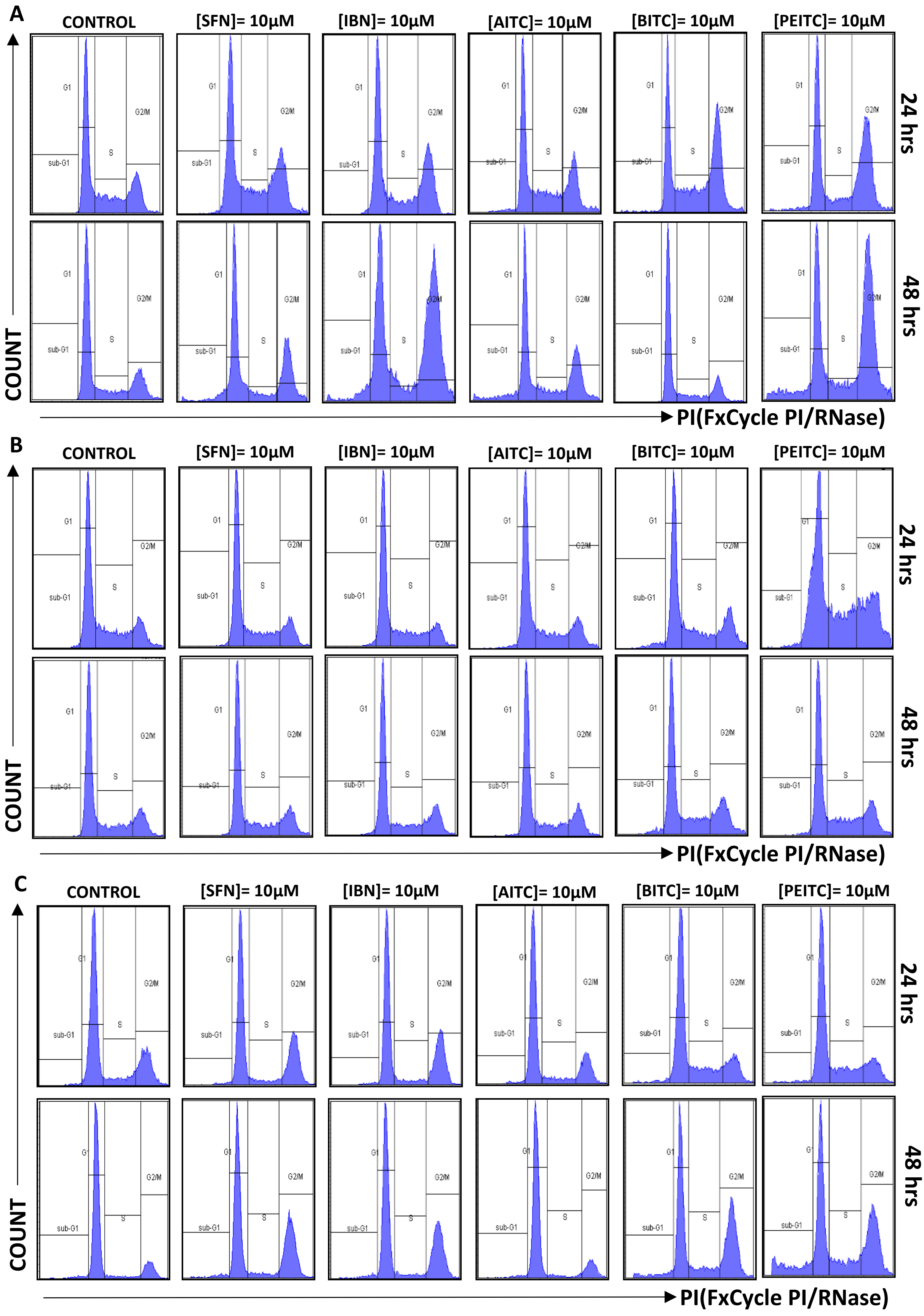
A
B
C
CONTROL
[SFN]= 10μM
[IBN]= 10μM
[AITC]= 10μM
[BITC]= 10μM
[PEITC]= 10μM
24 hrs
48 hrs
COUNT
PI(FxCycle PI/RNase)
G1
S
G2/M
sub-G1

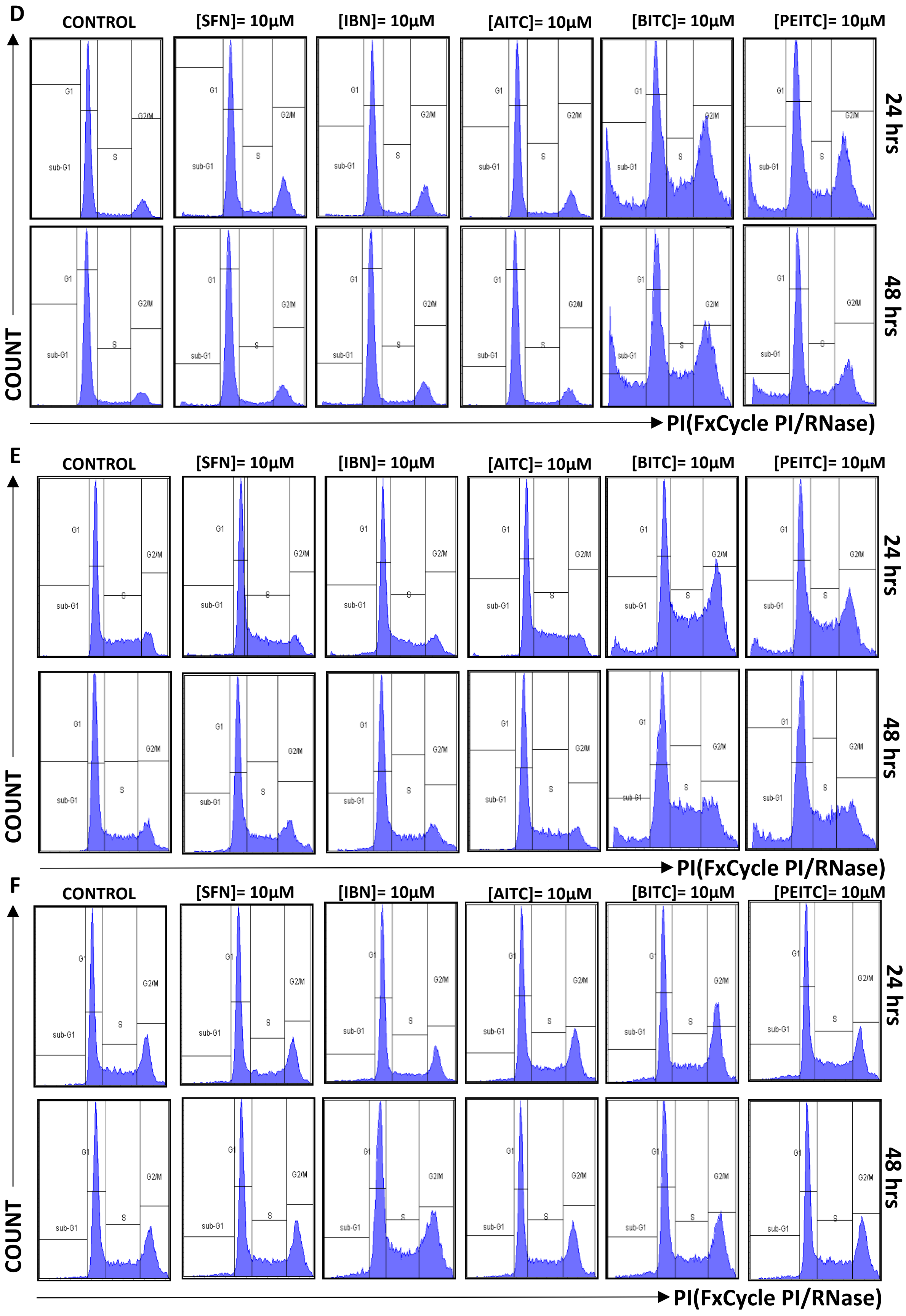

D
CONTROL
[SFN]= 10μM
[IBN]= 10μM
[AITC]= 10μM
[BITC]= 10μM
[PEITC]= 10μM
24 hrs
48 hrs
COUNT
PI(FxCycle PI/RNase)
G1
G2/M
S
sub-G1
E
CONTROL
[SFN]= 10μM
[IBN]= 10μM
[AITC]= 10μM
[BITC]= 10μM
[PEITC]= 10μM
24 hrs
48 hrs
COUNT
PI(FxCycle PI/RNase)
F
CONTROL
[SFN]= 10μM
[IBN]= 10μM
[AITC]= 10μM
[BITC]= 10μM
[PEITC]= 10μM
24 hrs
48 hrs
COUNT
PI(FxCycle PI/RNase)

Figure S3: The effect of ITCs (SFN, IBN, AITC, BITC, PEITC) on cell cycle in an *in vitro* model of malignant melanoma consisting of (**A**) human malignant melanoma (A375), (**B**) murine malignant melanoma (B16F-10), (**C**) human brain metastatic melanoma (VMM1), (**D**) human lymph node metastatic melanoma (Hs 294T), (**E**) human non-melanoma epidermoid carcinoma (A431) and (**F**) human immortalized keratinocyte (HaCaT) cell lines. Cells were seeded in a 100 mm dish and next day were exposed to 10 μM of each ITC for 24 and 48 hrs. Pre-fixed cells were stained with FxCycle PI/RNase fluorescent probe and the DNA content was determined by using a FACS Canto II flow cytometer (BD Biosciences, San Jose, CA, USA). All data were compared to the respective untreated (control) samples. Representative data from three independent experiments.

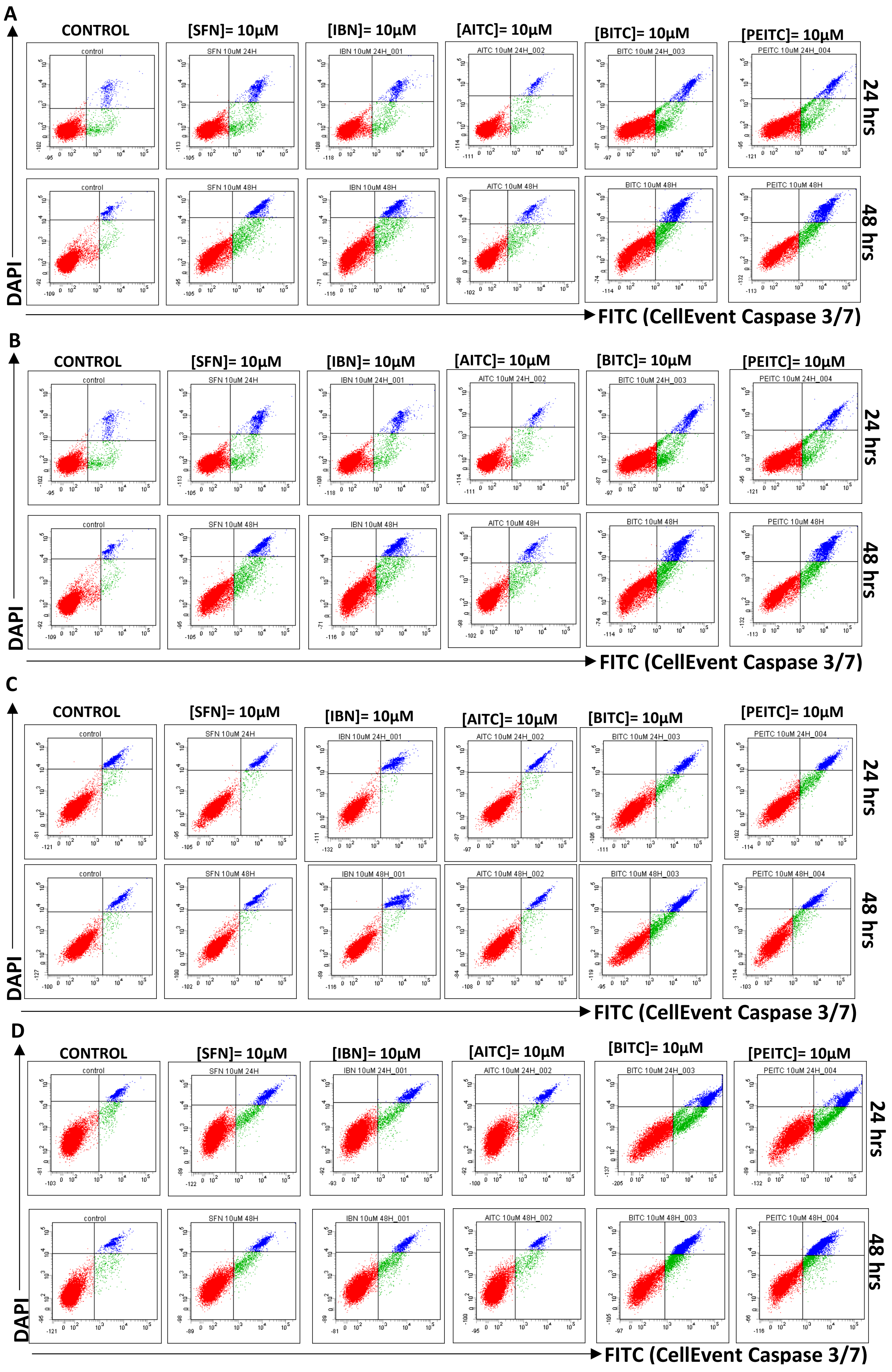
A
CONTROL
[SFN]= 10μM
[IBN]= 10μM
[AITC]= 10μM
[BITC]= 10μM
[PEITC]= 10μM
24 hrs
48 hrs
DAPI
FITC (CellEvent Caspase 3/7)
B
CONTROL
[SFN]= 10μM
[IBN]= 10μM
[AITC]= 10μM
[BITC]= 10μM
[PEITC]= 10μM
24 hrs
48 hrs
DAPI
FITC (CellEvent Caspase 3/7)
C
CONTROL
[SFN]= 10μM
[IBN]= 10μM
[AITC]= 10μM
[BITC]= 10μM
[PEITC]= 10μM
24 hrs
48 hrs
DAPI
FITC (CellEvent Caspase 3/7)
D
CONTROL
[SFN]= 10μM
[IBN]= 10μM
[AITC]= 10μM
[BITC]= 10μM
[PEITC]= 10μM
24 hrs
48 hrs
DAPI
FITC (CellEvent Caspase 3/7)

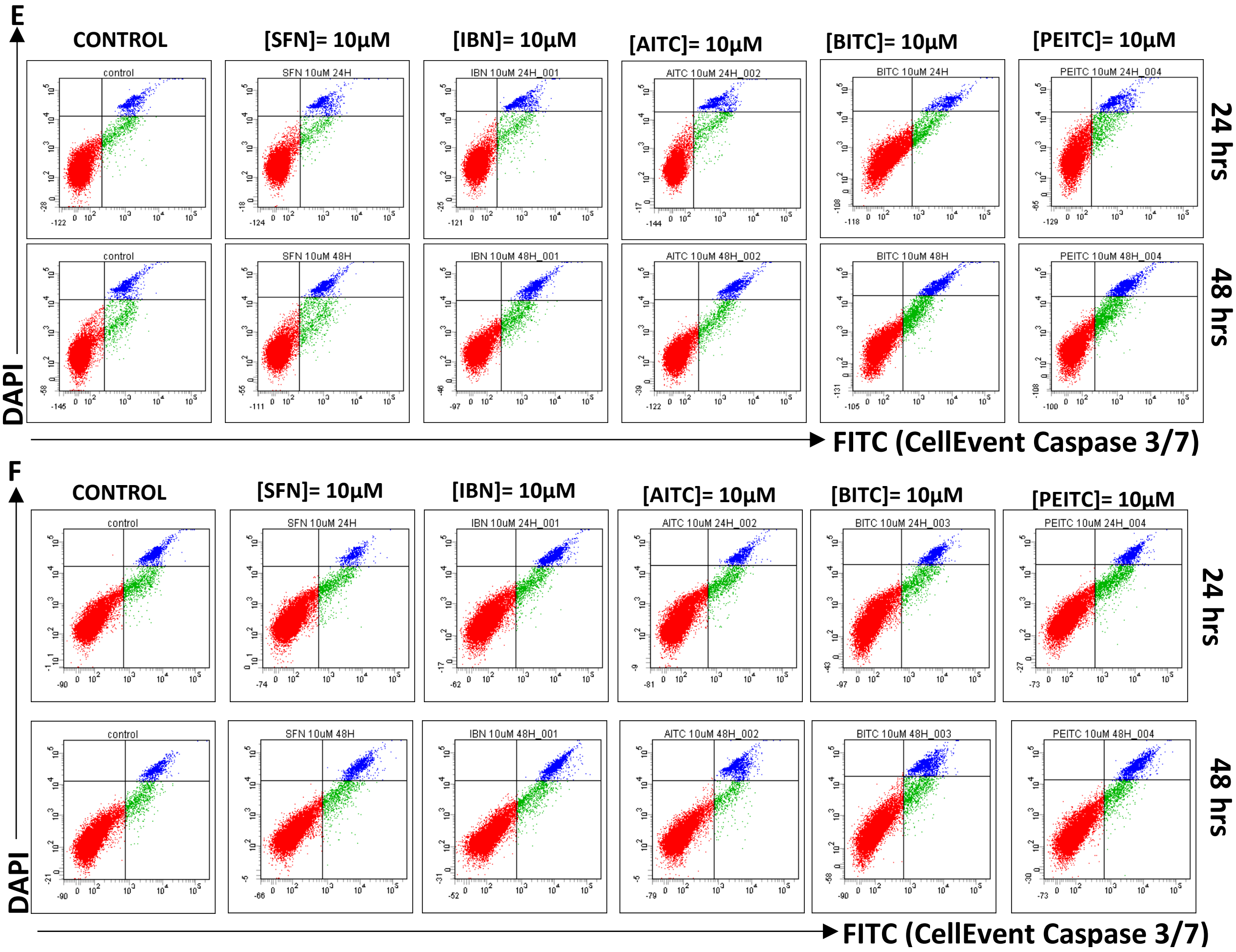

Figure S4: The effect of ITCs (SFN, IBN, AITC, BITC, PEITC) to induce apoptosis and/or necrosis in an *in vitro* model of malignant melanoma consisting of (**A**) human malignant melanoma (A375), (**B**) murine malignant melanoma (B16F-10), (**C**) human brain metastatic melanoma (VMM1), (**D**) human lymph node metastatic melanoma (Hs 294T), (**E**) human non-melanoma epidermoid carcinoma (A431) and (**F**) human immortalized keratinocyte (HaCaT) cell lines. Cells were seeded in a 100 mm dish and next day were exposed to 10 μM of each ITC for 24 and 48 hrs. Apoptotic and necrotic cell death was measured by the use of CellEvent Caspase 3/7 Green detection reagent and DAPI respectively. All determinations were made by using a FACS Canto II flow cytometer (BD Biosciences, San Jose, CA, USA). All data were compared to the respective untreated (control) samples. Representative data from three independent experiments.
